# The role of community advisory boards in community-based HIV clinical trials: a qualitative study from Tanzania

**DOI:** 10.1186/s12910-021-00737-w

**Published:** 2022-01-08

**Authors:** Godwin Pancras, Bruno F. Sunguya, Nathanael Sirili, Emmanuel Balandya, Eligius Lyamuya, Blandina T. Mmbaga

**Affiliations:** 1 Department of Bioethics and Health Professionalism, Muhimbili University of Health and Allied Sciences, P.O. Box. 65001, Dar es Salaam, United Republic of Tanzania; 2 Department of Community Health, Muhimbili University of Health and Allied Sciences, Dar es Salaam, United Republic of Tanzania; 3 Department of Development Studies, Muhimbili University of Health and Allied Sciences, Dar es Salaam, United Republic of Tanzania; 4 Department of Department of Physiology, Muhimbili University of Health and Allied Sciences, Dar es Salaam, United Republic of Tanzania; 5 Department of Microbiology and Immunology, Muhimbili University of Health and Allied Sciences, Dar es Salaam, United Republic of Tanzania; 6 Department of Pediatrics, Kilimanjaro Christian Medical University College, Kilimanjaro, United Republic of Tanzania; 7 Kilimanjaro Clinical Research Institute (KCRI), Kilimanjaro, United Republic of Tanzania

**Keywords:** HIV/AID clinical trial, Community Advisor Boards, Research ethics, Qualitative research

## Abstract

**Background:**

Community Advisory Boards (CAB) have become essential organs of involving communities in HIV clinical trials especially in developing countries. However, limited empirical evidence exists on the role of CABs in low and middle-income countries including Tanzania. This study aims at exploring the role of CABs in community-based HIV clinical trials conducted in Tanzania.

**Methodology:**

We adopted a phenomenological approach to purposefully select HIV clinical trial stakeholders. These included CAB members, researchers and Institutional Review Board (IRB) members in Tanzania. We conducted In-depth Interviews (IDIs) with ten participants and three Focus Group Discussions (FGDs) with eighteen participants. The data were thematically analyzed with the aid of MAXQDA software version 20.2.1.

**Results:**

The findings indicate that at every stage of implementation of a community-based HIV clinical trial, a functioning CAB is important for its success. This importance is based on contextualization of the informed consent process and protocol, managing rumours in the community, weighing trial risks and benefits, sensitizing the community, assisting participant recruitment, tracing and retention. However, being perceived as financial beneficiaries than community representatives emerged as a challenge to CAB members.

**Conclusion:**

The study empirically indicates the need for functioning CABs in every stage of implementation of community-based HIV clinical trials. The roles of which are interwoven in serving research goals and protecting the interests of the community and that of trial participants.

**Supplementary Information:**

The online version contains supplementary material available at 10.1186/s12910-021-00737-w.

## Introduction

The need for integration of community and participant’s voices into research cannot be overstressed [1, 2]. Over 30 years, community advisory boards (CABs) have formed a prominent mechanism through which this could be achieved [3]. Generally, CAB act as a guiding compass for researchers in understanding the cultural dimensions and needs of the researched community on one hand and voicing community concerns to researchers on another. This dual role of CABs promoting research goals while protecting community/participants interests further raises eyebrows [4]. Several research regulations and guidelines have been formulated mandating researchers to establish CABs as part of the community engagement mechanism. This emphasis has been echoed by international research funding organizations some of which include the Joint United Nations Programme on HIV/AIDS (UNAIDS) through its “Good participatory practice guidelines for biomedical HIV prevention trials” [5], and the U.S. National Institute of Allergy and Infectious Diseases (NIAID) through the Recommendations for Community Involvement in National Institute of Allergy and Infectious Diseases HIV/AIDS Clinical Trials Research report. As CABs continue to be emphasized and used as a means to protect research populations (thus in addition to ethics committees), their role has received less attention in low and middle-income countries including Tanzania.

CABs can be population-specific or broad community [6, 7]. The former comprise of people directly affected by HIV/AIDS such as people living with HIV, injecting drugs, caretakers and usually established for a specific study. Members of the broad community CAB come from diverse sections within the community and its members participate in different studies [8, 9]. The size of the CAB varies and largely depends on the protocol and researcher’s need, its members can range from as few as 8 to over 80 [7]. For Tanzania, the Ministry of Health recommends between 10 and 15 members one of which should be a representative from the research team for coordination [10]. This number is not legally binding lather it varies based on the discretion of the researcher. In this study, we aimed to explore the roles of CABs in community-based HIV clinical trials conducted in Tanzania.

## Methodology

### Design

The phenomenological qualitative study design was used to study the ethical role of CABs in HIV clinical trials in Tanzania. The design has been useful in gaining more understanding of the lived experience of the population affected by the phenomena under investigation [11]. More importantly, the design was accompanied by qualitative approaches to gain an in-depth understanding of the ethical role played by CABs. Unlike other research approaches, the qualitative approach by the constructivist paradigm enables researchers to be aware of different meanings that participants assign to their experience by answering how and why questions [12]. Furthermore, tailored semi-structured interview guides were used to elicit information from both in-depth interviews (IDI) and focus group discussion (FGD) participants.

### Study setting

The study was conducted in two Tanzania regions: Mbeya region located in the Southwest of Tanzania, and Dar es Salaam region in the Eastern part of Tanzania. The prevalence of HIV among people aged 15 years and above is 9.3% and 4.7% in Mbeya and Dar es Salaam region respectively [13]. The two regions have and continue to significantly participate in several HIV clinical trials [14–16]. In Tanzania, CABs came into existence between 2005 and 2007 at the time when the need to conduct community-based clinical trials including HIV clinical trials was on the rise [9]. Anecdotally, the National Health Research Ethics Committee (NatHREC) of Tanzania recommends community-based HIV clinical trials to establish and work in partnership with CABs. Most of the HIV clinical trials in Tanzania receive funding from foreign organizations which equally require CAB establishment for any grant awardees. Recently, CABs are becoming popular not only in HIV but also in non-HIV clinical trials including Tuberculosis.

For this study, members of its CAB were appointed by the research centre or local government leaders. Appointed members represented diverse groups within the community such as local leaders, religious leaders (Muslim and Christian), women, people living with HIV, female sex workers, police officers, teachers, youth, health professionals and groups with special needs. CAB membership lasted only 3 years and it could be renewed through a contractual agreement with the research centre. Moreover, CAB members had meetings scheduled once every four months or even more depending on the urgency. The responsibilities of the CAB were not limited to only HIV related trials but extended to other trials like Tuberculosis (TB), thus adopting the broad community model. The meetings would comprise CAB members together with the principal investigators or a research team representative. However, due to the Coronavirus disease (COVID19) outbreak some meetings had to be skipped. Members were not paid for being on the board but financially compensated for their time and travel expenses during meetings.

### Study participant

Participants of the study were divided into three groups. Group one: signified CAB members of an ongoing or previous HIV clinical trial. They are the ones who speak for the community and air out the concerns of the trial. Group two: Included local researchers or research coordinators of HIV clinical trials since they are the sole initiators and responsible for communicating research goals to the CAB and lastly, Group three: comprised of members of IRBs/ research ethics committees and are people responsible for reviewing and approving clinical trials on ethics grounds.

### Sampling procedure

Study respondents of all groups (I-III) were selected purposively depending on their experience and involvement in HIV clinical trial activities. Thus, purposive sampling deepens the understanding of how CABs play their role of protecting community and participant interests in HIV clinical trials by the identification of information-rich respondents [17]. The groups (I–II) were largely identified through research institutions and investigators of particular HIV clinical trials.

### Data collection

A total number of 28 stakeholders participated in a study of three FGDs and ten IDIs. CAB members were involved in FGDs whereas, researchers and IRB members were involved in IDIs as illustrated in Table 1. FGDs provided the best option for obtaining shared consensus among CABs about their role in HIV clinical trials. On the other hand, IDI was used to elicit depth information from researchers and IRB members due to their depth of understanding of HIV clinical trials and geographical dispersion. Pilot tested semi-structured interview guides were used as data collection tools (see Additional file 1). Moreover, two FGDs were divided based on gender and one comprised a mixture of both male and female respondents this was to reduce the effect of male–female patriarchal hierarchies existing in the community. With permission from respondents, a digital voice recorder was used to capture audio information. Field notes were also used to capture additional information that later informed the analysis. The interview began with a question on participants' demographic information followed by more general questions with probes. Interviews lasted between 17 and 76 min. Each FGD lasted 64 min on average. During data collection, respondents were free to decide the place of interview. All interviews were conducted in person using Swahili language but some IDI respondents used a mixture of Swahili and English to respond to questions. Data was collected for six months between August 2020 and February 2021. Data collection was stopped after attaining information saturation of the data being analyzed iteratively.

**Table 1:** Characteristics of included respondents

Description	IDI (researchers + IRB members)	FGD (CAB members)
Age range	29–63	25–67
Gender		
Male	5	8
Female	5	10
Total(n)	10	18
Research experience (years)	2–18	1–15 years. as a CAB member
Background	*Profession*	*Composition*
	Medical degree-7; Non-medical degree-3	Local leaders, government officials, health care professionals, representatives of people living with HIV, past HIV clinical trial participants, religious leaders, youth, police force, mass media, peer educators, and special groups

### Data analysis

Data was collected as part of large study on the ethical role of community advisory boards in HIV clinical trials. The collected text data was analyzed by researchers using the thematic analysis method which deployed both inductive and deductive reasoning techniques [18]. The first stage for data analysis began with the study team transcribing information gathered in audio format (by digital voice recorder) to text format. The transcription of recorded audio was aided by computer software named F4transkript version 3.03 [19]. Despite that, the independent researcher back-checked the transcribed audio and text data (transcript). Then it was followed by translating the transcripts from Swahili language (local language used for data collection) to English. The principal investigator made sure to back-check the translated transcripts. Before coding, the study team read the transcripts to familiarize themselves with the concepts portrayed by respondents. Then developed a coding plan [20]. During coding, codes were clustered into groups depending on their differences and similarities to form seven categories: contextualization of the study protocol and informed consent; managing community rumours; weighing risks and benefits; community sensitization; trial participant recruitment; tracing and retention of trial participants and CABs as financial beneficiaries. Two study team members coded the transcripts. It is important to note the whole process of data collection to analysis was conducted iteratively, that is after every two interviews data was analyzed before moving on to the next interview session. This ensured that emerging questions were incorporated into the interview guide to be further explored in the next interview. The whole process of coding was aided by Computer Assisted Qualitative Data Analysis Software named MAXQDA version 20.2.1.

### Ethical consideration

Ethical approval was obtained from the Senate Research and Publications Committee of the Muhimbili University of Health and Allied Sciences (MUHAS-REC-07-2019-04.E1). All study respondents provided individual informed consent voluntarily. Permission for audio recording was re-iterated to participants during interviews and how the collected data would be anonymously protected. During FGDs, respondents were instructed to use numbers when referring to fellow participants or themselves rather than names.

## Results

### Description of findings

#### Contextualization study protocol and informed consent forms

Respondents reported of CAB members played a crucial role in reviewing the trial protocol and mostly the informed consent forms before being administered to participants. That is, CAB members provided cultural and contextual insight about the study and helped to simplify the language written in protocol and informed consent forms. This was affirmed by a CAB member who reported that:

Before the study begins, they must bring the study documents and see if there is a problem, what is the safety for those who will be involved in the study what steps are being used to reach them, then, if we are satisfied all together then the study can continue (FGD1, Respondent 06)

Furthermore, unlike researchers, CAB members were expected to be more accustomed to the environment and cultural values of the potential participants and the community as a whole, therefore being in good a position to tailor the consent form and process to suit the local context. One of the CAB members said that:

…you know in every area there are traditions and customs so because I come from this community I know the language to be spoken and the manner in which you should speak… (FGD 2, Respondent 04)

However, when probed respondents reported being reluctant with the involvement of CAB members to directly administer the consent forms or even being present when a researcher is doing so. This is because their presence would breach confidentiality and interfere with the trial participant’s privacy. The following researcher noted that:

…I am the one asking for the consent from the participant... and all things related to the research I will tell him/her and not the CAB member. (IDI, Respondent 04)

One of the respondents who happened to be an IRB member proposed an exception, whereby CAB members could be invited when researchers are administering informed consent to participants. Said:

Unless you are doing a group consenting with them [CAB members] present ... but there is a very high risk of losing confidentiality and causing problems for those who are entering the study (IDI, Respondent 10)

### Managing community rumours

It was feared that the spread of rumours or false information in host communities could negatively impact an individual to consent to clinical trials especially to HIV vaccine trials. The rumours included trial participants being injected with active human immunodeficiency virus that causes AIDS, losing ability to reproduce, and fear of collected blood being used for something else or sold. However, the people who spread rumours within the community are health care workers and trial participants who are expected to be knowledgeable about the study science compared to the rest of the community. Circulating rumours elicited blame to CAB members since they are the ones who introduced researchers to the community. One researcher responded reported that:

…participants who come out of the study go to the street and spread information that researchers are infecting people with HIV… at the end of the day, they [community members] return the blame to the CAB” (IDI, Respondent 03)

Means used to alleviate rumours included educating the participants and the community through organized community gatherings as stated below by one of the CAB members:

…sometimes they call a press conference, or they can hold public meetings through village government officials to provide detailed education to citizens…” (FGD 2, Respondent 05)

### CAB’s ability to discern benefits and risks of a trial

The need to be aware of the risks and benefits of an HIV clinical trial was emphasized by IRB members, researchers and CAB members. However, scepticism arose to trust in CABs' ability to understand and appreciate the risks of the HIV clinical trial. Thus, CABs lag far behind in understanding the science of clinical trials which makes them unable to fully understand the inherent benefits or risks of the HIV clinical trial. CABs assumed that researchers and IRB members knew better when it comes to understanding the risks and benefits of the trial, as it was reported by one of the CAB members during FGDs:

The issue of recognizing the risks of research is difficult because we are not scientists” (FGD 2, Respondent 01)

In contrast, it was expected by researchers and IRB members that CAB should be able to understand and conversantly communicate risks and benefits back to their respective communities. As noted by one of the IRB members below:

Once they [CAB members] have identified the benefits and risks of the study, they will help to inform those potential participants that this study is beneficial… or has risks. (IDI, Respondent 09)

In addition, the above respondent advised that, for CABs to be competent in discerning risks and benefits, it is important the membership composition include knowledgeable individuals. As reported below by an IRB respondent:

It depends on the community and the composition of the CABs ... there should be people with an understanding that joining this study has these benefits and these consequences... (IDI, Respondent 09)

### The extent of CAB's involvement in weighing risks and benefits of a trial

Respondents reported the importance of fully sharing information regarding trial benefits and risks with CAB members because they represented both the community and potential participants. And if participants are harmed in any way during the trial, CAB members could be held accountable. One of the CAB members said that:

It is very important for us to be involved and to know what is going on so that if there is a problem in the community, I know how to solve it… (FGD 3, Respondent 01)

In addition, it was reported that sometimes CABs challenged researchers on what ought to be the benefits of the respective trial. Having interests and views contrary to the known global standards was reported by an IRB respondent as being the source of differing perspectives regarding benefits and risks:

…they were [CAB members] with a completely different view of the study and its benefits so we found ourselves arguing over the benefits of the study… because, I would say, of different interests. (IDI, Respondent 08)

However, CAB members could not hide from the questions posed by participants and the community about the benefits of the trial they are participating in. One CAB respondent asserted that:

....he participated in this kind of research [HIV clinical trial] but later when the results and achievements are realized how does he/she benefit…those are the questions we [CAB members] face (FGD 2, Respondent 05)

### Community sensitization

During data collection, respondents reported about CAB's involvement in sensitization events of HIV clinical trials. During this period, the community is informed about the presence of researchers and the kind of trials going to be conducted. This is done either individually by CAB members or side-by-side with researchers. The reason behind CABs involvement in sensitization as testified by one of the researchers was:

The importance of the CAB is to encourage participants to participate in immunization [vaccine] research using real examples of current CAB members who were previous participants… (IDI, Respondent 07).

However, in so doing CAB members refrained from the use of coercive language when communicating with communities and potential participants instead they emphasized more on the willingness of those who want to participate in the trial. CAB members did not have to wait for large community meetings to convey research information instead they did so even with people gathering in small groups. One respondent who is a CAB member said that:

…if I have been fortunate to meet those girls who are in the prostitution business…they have come in front of me three, four or five I take that opportunity without waiting to tell them a few words about the vaccine trial (FGD 3, Respondent 05)

### Trial participant recruitment

During the selection of eligible participants for the HIV clinical trial, CAB's role was also extrapolated by the respondents. CAB members acted as an entry point to researchers recruiting participants from communities or groups they represent. Depending on the situation and the kind of participants needed, CABs also acted as advisors on the best locations to find potential participants. One of the researchers alleged that:

…you will tell him the characteristics of the people you want, so he will look in his/her [CAB member] area where those people [participants] are located... (IDI, Respondent 01)

The situation becomes even more challenging when researchers want to recruit non-HIV participants or healthy volunteers, who normally do not visit HIV care and counselling clinics. The best way to get such participants was through coordination with CABs as it was reported by one of the researchers below:

…for infected people [people living with HIV], it is easy to get them because they have their clinic but those who are negative it is a little work… through them [CAB members] have helped us find these people who are not infected [with HIV]. (IDI, Respondent 03)

Still, there was an agreement among respondents that CAB members cannot decide who should or should not participate in the HIV clinical trial lather researchers are the ones in a good position to do so. Even researchers themselves have to appeal to scientific reasons and methodology when choosing participants. During focus group discussions one of the CAB members stressed that:

The CAB cannot say that he/she should not participate while researchers want him/her to participate in research (FGD 1, Respondent 07)

Similarly, one of the researchers cemented that:

CAB, once they are told that this is our target… their job is over, they should not go and say that this one enters the treatment group and this one in the control group (IDI, Respondent 09)

### Tracing and retention of trial participants

With the fear of losing participants during HIV clinical trials, CABs turned to be helpful to researchers in terms of tracing and retention of participants. Most CAB members were aware of the geographical area that participants were recruited from. Also, some of them were local leaders who would easily communicate with other community members who happen to know the participant. The use of CABs in tracing of participants needed to be specified during protocol preparations. CABs came into play when researchers could not retain or trace participants on their own despite the risk of confidentiality. One of the respondents who is a researcher revealed that:

…we do not go directly to CAB members... because when a participant comes to us we have consent form between us [researcher and participant]... but when cannot be reached and we have no other place to find him/her, then the CAB helps. (IDI, Respondent 03)

When CAB members are called upon, they start by contacting local leaders and after which they communicate back to the researchers regarding the availability status of the participant. One of the CAB members said:

Our job is to do tracing…from which local leader [catchment area] was he/she [the participant] coming from so that I can go and tell [researchers] this guy has moved from this area go somewhere you will find him (FGD 2, Respondent 04)

### CAB members as financial beneficiaries

CAB members reported being perceived by the community and groups they represent as financial beneficiaries. This was contributed by how smart CAB members appear and behave in the community. Also, using the word ‘board’ member was reported to be directly associated with government boards or non-research boards whose members are assumed to be highly paid as reported by one of the CAB members below:

When someone says I am on board [CAB] they know you are a person earning money because the word ‘board’ is not a joke…someone may even come and ask you for money. (FGD 2, Respondent 01)

In addition, it was said that trial participants would compare the amount they are reimbursed to participate and what CAB members are likely to get paid. One of the CAB members noted that.

…the participant may have read the consent form and sees that he gets 15000Tshs [6.5USD] as transport fare now he thinks if I the participant gets this amount, the CAB member earns millions. (FGD 3, Respondent 02)

This led to a sense of mistrust due to an assumption that whatever CABs are doing or telling the community it is because they are paid by the research team and not for the community interest. Trying to change this notion ingrained within the community, CAB members had to genuinely explain themselves about not receiving any financial gains from the research team instead they are volunteering. Yet in the meantime CAB members indicated the need for increased allowances per seating near to how other ‘boards’ are being paid. One CAB member said that:

… as an advice, policymakers if they can, to improve CAB’s allowance rates not to be too low even though it is voluntary… (FGD 3, Respondent 7)

## Discussion

The role of CABs in HIV clinical trials especially in low and middle-income countries cannot be underrated. During this qualitative study, HIV clinical trial stakeholders reported thorough involvement of CAB members in reviewing study materials, managing community rumours, weighing risks and benefits of the trials, sensitizing the communities for participation in trials as well as assisting in participant recruitment and retention process. But, CABs members being termed as financial beneficiaries was pointed out as a challenge.

Engaging CABs in the informed consent process included reviewing study protocol and informed consent forms. Informed consent is among the requirements of ethical conduct of research [21]. It is through the informed consent process that the rights of trials participants are respected. An appropriate informed consent process assures that a participant fully understands the study and willingly opts to or not to participate [22]. Throughout this study, CABs reported having reviewed the consent form first before being administered to trial participants. The involvement assisted researchers discover the social and cultural suitability of their consent forms. Equally, a study conducted by Mwinga and Moodley in Zambia reported of 10 out of 14 interviewees identified the consent form and protocol review as an important CAB role [23]. These findings also affirm what Strauss and colleagues suggested about initiatives towards involving communities in the informed consent process recommending CAB involvement as one of the methods [24]. With that in mind, it is clear that the traditional researcher-participant consent process should be enhanced by CABs, especially for community-based HIV clinical trials. However, caution should be taken so that CABs do not infringe the rights, privacy and confidentiality of trial participants whatsoever during the consenting process.

In addition to that, CABs also have a crucial role in managing rumours arising in HIV clinical trials. This is not surprising especially to clinical trials recruiting participants from the community. In this study, rumours ranged from stealing blood for sale, injecting live HIV to causing dysfunction of reproductive organs. According to Gordon Allport and Leo Postman, people spread rumours because of the uncertainty of what is going on and anxiousness for the answers [25]. Employment of CABs whose members are fully aware of the trial and can communicate in a language that is easily understood to the community helps alleviate the rumours. An empirical study conducted in Los Angeles, warned about the spread of rumours about HIV vaccines, especially when people have limited information and knowledge on how they work [26]. If rumours remain unattended, most community-based HIV clinical trial activities could be halted.

Apart from contextualizing the informed consent process and managing community rumours, the CAB’s role in weighing risks and benefits of the trial was also an important element worth exploring. Weighing the risks and benefits of a trial is a very critical task that has been dedicated uniquely to research ethics committees [27, 28]. In most developing countries, most research institutions still lack ethics committees and those that have, are faced with inadequate resources and less experienced staff to ethically review the risks and benefits of clinical trials [29]. For that reason, an appeal to CAB members to act as an additional layer to weighing the risks and benefits of clinical trials turn out to be paramount. Unsurprisingly, our findings indicate that CAB members could be ill-prepared and hesitant to effectively take on such responsibilities just because they fall short of scientific and ethics literacy. That being a case, respondents have argued for CABs to at least comprise knowledgeable members to adequately discern the risks and benefits of HIV clinical trials. This is similar to a study conducted by Shubis and colleagues in Tanzania which stressed the need for literate CABs members to represent the community in research [9]. Thus an assumption could be made that a literate CAB could avoid conflicts with international research ethics standards and/or researchers. However, some scholars have identified that even the health-literate CAB members still face difficulties in understanding and communicating study scientific information including risks and benefits [30]. Further research is needed to understand how CAB interests help define risks and benefits as opposed to what is stipulated in international ethical guidelines.Similarly, the role of CABs in selecting trial participants was well explored. It includes CAB’s involvement in community sensitization, trial participant recruitment, and tracing and retention of trial participants. For community sensitization, it is during this time that the whole community is informed about the trial and what it involves. It is during this time that CAB members, not researchers, understand what the community wants to hear, and what mechanisms should be used to deliver the message. In this study, instilling community trust in HIV clinical trials was perpetuated by CAB members. Siyabonga Thabethe and colleagues reported similar findings whereby community members showed trust in information coming from someone they are familiar with or someone unaffiliated to the study site or researchers [31]. Therefore, during HIV clinical trial community sensitization activities, involving CAB members would tremendously invite trust from the host community and potential participants.

In addition, the findings indicated that CAB members’ role in the recruitment process is more advisory than having a final say. They could advise researchers on where to get potential participants and on how to approach them since they are more acquainted with the geographical area and values of the community they represent. This in turn easy the recruitment process by making sure potential participants are timely located and enrolled into HIV clinical trials. It has been reported that delays (from bench to bedside use of the investigational product) encountered in most clinical trials are attributed to faults in the recruitment process, and the way out of this, is to equally involve all stakeholders prior to recruitment [32]. For that reason, CAB members should not be left out in the recruitment process, instead, are to be treated as equal partners (their opinion matters) when preparing a recruitment plan. Nonetheless, CAB members are not to be used to decide who should or should not participate or to which experimental arm participants should be allocated.

CABs role in tracing and retention of trial participants hugely contributed to attaining the required number of clinical trial participants. When researchers failed to reach their trial participants either physically or by phone calls they appealed to CAB members for help. Since most CAB members are local leaders or well-known people within the community, they can easily trace participants using their social networks. This is advantageous to researchers and could reduce the number of lost to follow-ups that would statistically disqualify or prolong the clinical trial. Similarly, Bahemuka and colleagues found that tracing and retention of potential HIV vaccine trial participants could be improved through social networks established by CABs and other stakeholders in the community [33]. In addition, a study conducted by Kiwanuka and colleagues about tracing methods of HIV vaccine potential participants reported of the physical contact tracing method (using community health mobilizers) having less than 83% participant retention after a follow-up period of 18 months [34]. Therefore, it is clear that CABs should be integrated into HIV clinical trials as one of the techniques to enhance tracing and retention of trial participants which has long been problematic. However, more investigation is still required to understand whether the use of CABs to trace and retain participants could jeopardize the prior researcher-participant agreements of maintaining confidentiality.

Despite the foregoing roles, CABs reported being assumed by the community they represent as financial benefitiaries . The word ‘board’ exacerbated this notion because, in Tanzania, boards are popularized as government-linked entities comprised of experts with a government-driven mission. This is different from South Africa where the word ‘board’ meant having legal authority and power to make decisions [4]. Also, unfound notion exists that board members usually receive higher allowances whenever they have meetings. To overcome this misconception we propose the word ‘group’ instead of ‘board’ be used, that is community advisory group (CAG). The term CAG is slightly gaining popularity and has increasingly been used in some articles to refer to CAB [30, 35]. Additionally, the findings from this study add to an increasing dilemma of providing incentives or remuneration to CAB members and the risk to compromise their role [23]. However, our findings indicate that CAB’s role may be compromised due to misconceptions held by the community regarding reimbursement and transport fare offered to CAB members. The problem of which could be resolved by creating an independent CAB as suggested by Nyirenda et al. [30], but to be avoided altogether, there should be transparency in financial transactions between the CAB and research team.

### Methodological considerations

We discuss methodological consideration under two parts, the study limitations and trustworthiness. Regarding study limitations, first: this study used a qualitative approach whose information cannot be generalized or applied in a different context. Interviewees shared their insights and experiences that could not necessarily be similar to those who did not take part in this study. However, to overcome this limitation, interviews were conducted with different groups and in two regions of Tanzania. Second: At the time of the study only one CAB participating in HIV clinical trials was active and had over 15 years of experience. Other CABs were dismantled after trial completion and member weren’t accessible. Having perspectives from other CABs could have broadened our understanding about the role of CABs in the country. Lastly, views from the community being represented were not captured in this study thus making it difficult to ascertain the CAB’s role to the community. On the other hand, to ensure trustworthiness, an appeal was made to the four Guba’s criteria [36]. To ensure transferability, the study included a detailed description of the methodology including study settings and study population. Credibility was attained through triangulation whereby diverse groups of respondents (Researchers and IRB members) from different study sites, institutions and regions were recruited: in Dar es Salaam and Mbeya region. The same goes for the use of both FGDs and IDIs to explore the role of CAB in HIV clinical trials. Moreover, results and interpretations were presented to an independent competent qualitative researcher for further review to ensure the dependability of our findings and the study as a whole. For confirmability, researchers shared the synthesized results with a few participants to ensure that their voices are reflected in the study and reduce the researcher's bias. Lastly, transferability to other similar contexts has been ensured by providing depth description of the study settings and respondents.

Besides, further research into this area is hailed not only in HIV but also in non-HIV clinical trials looking at ethical, social and motivational implications behind CABs. But also studies exploring the CAB’s role based on perspectives of communities being represented are highly encouraged.

## Conclusion

The findings indicate the crucial roles of CABs in implementation of community-based HIV clinical trials in low and middle-income countries like Tanzania although most of them seem to aid the research team and research process. Moreover, misconception about the CAB members being the financial beneficiaries from the community-research team interaction risks hampering the mentioned roles. Overall, the role of CABs is to protect community and participant interests while advising researchers on how their research goals could be attained.

## Supplementary Information

**Additional file 1.** **Appendix 1:** Focus group discussion (FGD) guide for CAB members. **Appendix 2:** Individual interview (IDI) guides for researchers/research coordinators. **Appendix 3:** Individual interview (IDI) guide for IRB members.

## Data Availability

The datasets used and/or analysed during the current study are available from the corresponding author on reasonable request.

## Abbreviations

CABs: Community Advisory Boards
CAG: Community Advisory Group
FGDs: Focus Group Discussions
IDI: In-depth interviews
HIV: Human immunodeficiency virus
