# Supplementary material for: The role of community advisory boards in community-based HIV clinical trials: a qualitative study from Tanzania
Source: BMC Med Ethics. 2022 Jan 8;23:1. doi: 10.1186/s12910-021-00737-w (PMC8741593; doi:10.1186/s12910-021-00737-w)
Supplement: Supplementary file 1 — Additional file 1. Appendix 1: Focus group discussion (FGD) guide for CAB members. Appendix 2: Individual interview (IDI) guides for researchers/research coordinators. Appendix 3: Individual interview (IDI) guide for IRB members. [file 12910_2021_737_MOESM1_ESM.docx]

## Appendix 1: Focus group discussion (FGD) guide for CAB members [English version]

**Introduction:** Thank you very much for agreeing to participate in this discussion. As CAB members you have been selected because you have been representing the community in HIV clinical trial (s) in your village/town/street/hamlet. During our discussions, there are no wrong answers; all answers are accepted so kindly feel free. I would like to remind you that our discussion may take 1 to 2 hours, and it will be audio recorded to grasp points that could be difficult to write down but also for easy analysis of the information later on. **Please do use the numbers provided to identify yourself or address another member during our discussion.**

**For Demographics**: A simple registration form with serial number for each individual will be used to collect information on their age, marital status, education level, length of time as a board member (or resident of the village/hamlet/town)(It will be prepared at the field depending on the number of participants)

**Preliminary information**

I would like us to start by everyone introducing him/her self (use a given number instead of your name).

a) When was your CAB established? ………..

b) How often do you meet? …………

**Qn1**. Generally, what are your responsibilities as CAB members in HIV clinical trials?

1. How are you involved in the informed consent process/ Or How should you be involved in the informed consent process of HIV clinical trials?
2. What information do researchers share with you as CABs members?/What information should researchers share with you as CAB members?
3. Have you ever asked researchers to change their informed consent form or process? Why so?

Qn2. Can you explain to us how you interact with community members?

1. How do you approach them
2. What methods do you use
3. Can you tell us one real life scenario of how you interacted with the community in the previous HIV clinical trial
4. How does the community think of you?

**Qn3**. What were/are the risks of HIV clinical trials your community participated?

1. How do you identify the risks of an HIV clinical trial? And how do you address them?
2. Do you think you should be involved with researchers in identifying and resolving risks? Why?

**Qn4**. What were/are the benefits of HIV clinical trial your community participated?

1. How are you able to identify the benefits of an HIV clinical trial?
2. Do you think you should be involved in deciding the benefits of an HIV clinical trial? How?

**Qn5**. How can you as CAB members weigh the risks and benefits of an HIV clinical trial that your community is participating/ participated?

1. Should you be involved in balancing the risks and benefits of an HIV clinical trial? Why?

**Qn6**. What role(s) do you have as CAB members when the research team is recruiting participants for HIV clinical trial?

a) How should the CAB be involved in the participant recruitment process for trial?

b) How can the CAB ensure fair (unbiased) recruitment of participants?

**Qn7: What challenges do you as CAB members face when implementing your activities in HIV clinical trials?**

**Closure of the interview**: Is there anything else that you would like to share with us about what we have just discussed?

Thanks!

## Appendix 2: Individual interview (IDI) guides for researchers/research coordinators [English version]

**Introduction:** Thank you very much for agreeing to participate in this interview. You have been selected among other members because you have experience and knowledge in researching /coordinating CAB for an HIV clinical trial. During this interview there are no wrong answers, all answers are accepted so kindly feel free. I would like to remind you that this interview may take 30 to 60 minutes and it will be audio recorded to grasp points that could be difficult to write down but also for easy analysis of the information later on.

**Qn1. Can you share with me your experience with CABs in HIV clinical trials?**

**Qn2**. Generally, what are your responsibilities towards the CAB in an HIV clinical trial?

1. What information do you share with CAB members? / What information should researchers share with CAB members?
2. Should CABs be involved in the informed consent process of HIV clinical trials? Why?
3. Has the research team ever re-designed/changed the informed consent form or process to due to CAB views about the trial? Please tell me more.

**Qn3**. Should CABs be involved in weighing the risks and benefits of an HIV clinical trial? Why?

- How can the CAB be used to weigh the risks and benefits?

**Qn4**. What role(s) does the CAB have when the research team is recruiting participants for an HIV clinical trial?

- Do you think the CAB should be involved in deciding which participants to participate in the trial? Why so?

**Qn5**. What challenges do CABs face in HIV clinical trials?

- How can CABs be empowered?

**Closure of the interview**: Is there anything else that you would like to share with me about what we have just discussed?

Thanks!

## Appendix 3: Individual interview (IDI) guide for IRB members [English version]

**Introduction:** Thank you very much for agreeing to participate in this interview. You have been selected among other members because you have the experience of participating HIV clinical trial ethical reviews. During this interview there are no wrong answers, all answers are accepted so kindly feel free. I would like to remind you that this interview may take 30 to 60 minutes and it will be audio recorded to grasp points that could be difficult to write down but also for easy analysis of the information later on.

Preliminary information:

| a | Gender |  |
| --- | --- | --- |
| b | Age |  |
| c | Education level |  |
| d | Work experience (years) as an IRB member |  |
| e | Profession |  |
| f | Number of HIV clinical trials reviewed/assessed |  |

**Qn1. Generally, what is your experience with HIV clinical trials involving community advisory boards (CABs)**

1. Should IRBs work together with CABs? Why so? How can that be done?
2. What is the responsibility of CABs in HIV clinical trials that you know of?
3. Should CABs be involved in the informed consent process of HIV clinical trials? Why? How?

**Qn2**. Should CABs be involved in weighing the risks and benefits of an HIV clinical trial? Why?

- How can the CAB be used to weigh the risks and benefits?

**Qn3**. What role(s) should the CAB have when the research team is recruiting participants for an HIV clinical trial?

- Do you think the CAB should be involved in deciding which participants to participate in the trial? Why so?

**Qn4**: What challenges do CABs face in HIV clinical trials?

- How can CABs be empowered?

**Closure of the interview**: Is there anything else that you would like to share with me about what we have just discussed?

Thanks!
